# Autocrine Production of Interleukin-34 Promotes the Development of Endometriosis through CSF1R/JAK3/STAT6 signaling

**DOI:** 10.1038/s41598-019-52741-1

**Published:** 2019-11-14

**Authors:** Kaiqing Lin, Junyan Ma, Yaomin Peng, Meina Sun, Kaihong Xu, Ruijin Wu, Jun Lin

**Affiliations:** 1 Department of Gynecology, Women’s Hospital, Zhejiang University School of Medicine, 1 Xueshi Road, Hangzhou, Zhejiang 310006 People’s Republic of China; 2 Key Laboratory of Reproductive Genetics (Zhejiang University), Ministry of Education, 310006 Hangzhou, People’s Republic of China

**Keywords:** Growth factor signalling, Infertility

## Abstract

Interleukin (IL)-34 plays a critical role in cell proliferation, differentiation, apoptosis, angiogenesis, inflammation and immunoregulation. Numerous diseases can be attributed to the dysregulation of IL-34 signaling. This study was performed to investigate the function of IL-34 in the pathogenesis of endometriosis. Firstly, by enzyme linked immunoabsorbent assay, we found that IL-34, VEGF, MMP-2 and MMP-9 were increased in the sera of patients with endometriosis. Secondly, exposure to IL-34 promoted the proliferation, migration and invasion of eutopic endometrial stromal cells (ESCs). Additionally, stimulation with IL-34 up-regulated colony-stimulating factor 1 receptor (CSF1R), p-JAK3, p-STAT6, VEGF, MMP-2 and MMP-9 in these eutopic ESCs. Treatment with AS1517499, an inhibitor of STAT6, remarkably abrogated the alterations induced by IL-34. A Chromatin immunoprecipitation (ChIP) assay demonstrated binding of STAT6 to the IL-34 promoter, further implicating STAT6 in IL-34 signaling. Notably, reverse results were obtained in ectopic ESCs with the application of an IL-34 neutralizing antibody. *In vivo*, AS1517499 suppressed the maintenance of endometriosis lesions in rats. In summary, autocrine production of IL-34, mediated by STAT6, promoted the development of endometriosis *in vitro* and *in vivo* through the CSF1R/JAK3/STAT6 pathway. Our research reveals the function of IL-34 in endometriosis, which may provide insight into novel therapeutic strategies for endometriosis.

## Introduction

Endometriosis, a gynecological disease in which endometrial tissues are found outside the uterine cavity, such as on the ovary and pelvic peritoneum, commonly occurs in women of reproductive age^1–3^. Pelvic pain, dysmenorrhoea, dyspareunia, difficulty to defecate, and infertility are the most common complications of endometriosis and seriously affect the quality of life in patients^4,5^. Moreover, due to the misinterpretation of symptoms and the lack of potent early diagnosis strategies, endometriosis diagnosis is often delayed by close to ten years^6,7^. Pain relief is the primary purpose of treatment. Pharmacotherapies, such as the application of oral contraceptives or gonadotropin-releasing hormone agonists, and surgical resection of the endometriosis lesions are the main treatment options. However, progestin resistance and relapse greatly restrict the efficacy of these treatments^2,8^.

Endometriosis is also an estrogen-dependent inflammatory disease^9^. Mounting research suggests that cytokine-mediated innate immunity is involved in the pathogenesis of endometriosis^10–12^. For instance, interleukin (IL)−17 facilitated the formation and persistence of endometriosis lesions by increasing angiogenesis and the secretion of pro-inflammatory cytokines^13^. IL-34 is a newly discovered cytokine regulating the differentiation and proliferation of monocytes and is an alternative ligand of colony-stimulating factor 1 receptor (CSF1R)^14,15^. The engagement of IL-34 induces the phosphorylation and activation of CSF1R, which subsequently triggers signaling cascades that are associated with cell proliferation, differentiation, apoptosis, angiogenesis, inflammation and immunoregulation^16–19^. In the present study, through reanalysis of the expression profile of IL-34 in the microarray data of the Gene Expression Omnibus (GEO) database (GSE51981)^20^, we found that IL-34 was increased in clinical samples with endometriosis. However, the effect of IL-34 in endometriosis has not been elucidated. Thus, we hypothesized that IL-34 may play an important role as a multifunctional inflammatory cytokine in the pathogenesis and persistence of endometriotic lesions.

## Results

### IL-34 is increased in endometriosis

**Figure 1:**
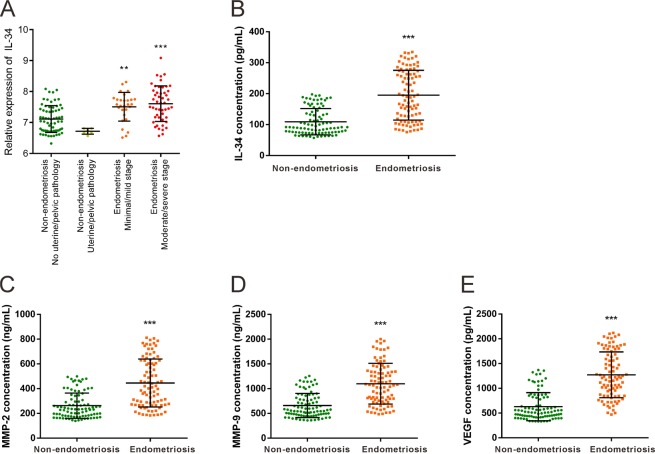
IL-34 is increased in endometriosis. (**A**) The differential expression of IL-34 in endometrial specimens obtained from the Gene Expression Omnibus (GEO) database (GSE51981). (**B**–**E**) The secretion of IL-34 (**B**), MMP-2 (**C**), MMP-9 (**D**) and VEGF (**E**) in the serum of endometriosis patients (n = 90) and non-endometriosis patients (n = 90) admitted at Women’s Hospital, Zhejiang University School of Medicine as detected by ELISA. **P < 0.01, ***P < 0.001.

Considering the crucial role of IL-34 in numerous disease states, we investigated the differential expression of IL-34 in endometrial samples with or without endometriosis using the microarray data in the Gene Expression Omnibus (GEO) database (GSE51981)^20^. As shown in Fig. 1A, IL-34 expression is increased in endometriosis tissues at all stages (Minimal/Mild, Moderate/Severe), implicating the role of IL-34 in the pathogenesis of endometriosis. We further collected the sera of endometriosis patients to confirm the changes in IL-34 expression levels. Results demonstrated that IL-34 secretion was elevated in endometriosis patients (Fig. 1B). Moreover, we also detected the serum levels of proteins associated with cell proliferation, angiogenesis, and epithelial-mesenchymal transition processes which are closely related to the pathogenesis of endometriosis, such as VEGF, MMP-2 and MMP-9,^21–23^, by ELISA. The results showed that VEGF, MMP-2 and MMP-9 were elevated in the sera of the patients suffering from endometriosis (Fig. 1C–E). These results are consistent with previous findings^21–23^.

### IL-34 potentiates the proliferation, migration and invasion potential of eutopic ESCs

**Figure 2:**
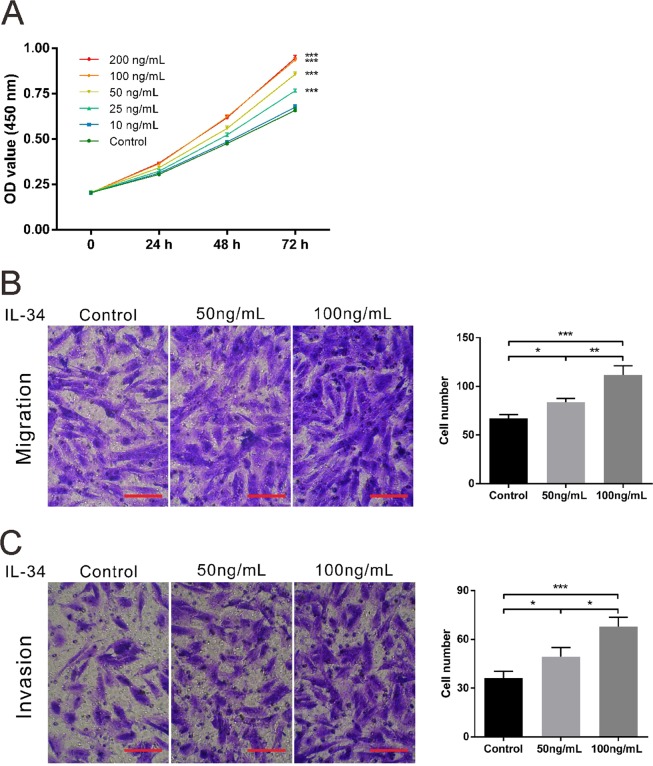
IL-34 promotes the proliferation, migration and invasion of eutopic ESCs in endometriosis. Eutopic ESCs were isolated from five individuals and three replicates were taken for each experiment. Representative results are shown. (**A**) The effect of recombinant IL-34 on ESC growth was detected by CCK-8 assay. (**B**,**C**) Left panels are representative images of ESC migration and invasion when stimulated with recombinant IL-34 (50 and 100 ng/mL). Right panels are a quantification of the number of migrated and invaded cells. Scale bar: 100 μm. *P < 0.05, **P < 0.01, ***P < 0.001.

Next, eutopic ESCs were isolated and immunohistochemical (IHC) staining for CD10, a specific surface marker for endometrial stromal cells^24^, and CK19, a specific marker for epithelial cells, was performed to identify ESCs. A strong, positive expression of CD10 was detected in the isolated cells, whereas CK19 was almost undetected (Fig. S1). These data suggest a successful isolation of ESCs. Then, to detect the function of IL-34 *in vitro*, eutopic ESCs were treated with increasing doses of recombinant human IL-34 (0–200 ng/mL). We found that IL-34 potentiated eutopic ESC growth in a dose-dependent manner at doses of 25 to 100 ng/mL (Fig. 2A). The proliferation rate of ESCs was similar at 100 ng/mL and 200 ng/mL doses of IL-34, thus doses of 50 ng/mL and 100 ng/mL were used in the following transwell assay. As indicated in Fig. 2B,C, IL-34 also enhanced migration and invasion in a dose-dependent manner. Taken together, elevated IL-34 expression may promote eutopic ESC proliferation, migration and invasion to facilitate the development of endometriosis.

### CSF1R/JAK3/STAT6 activation and IL-34 autocrine production are responsible for the increased proliferation, migration and invasion of eutopic ESCs

**Figure 3:**
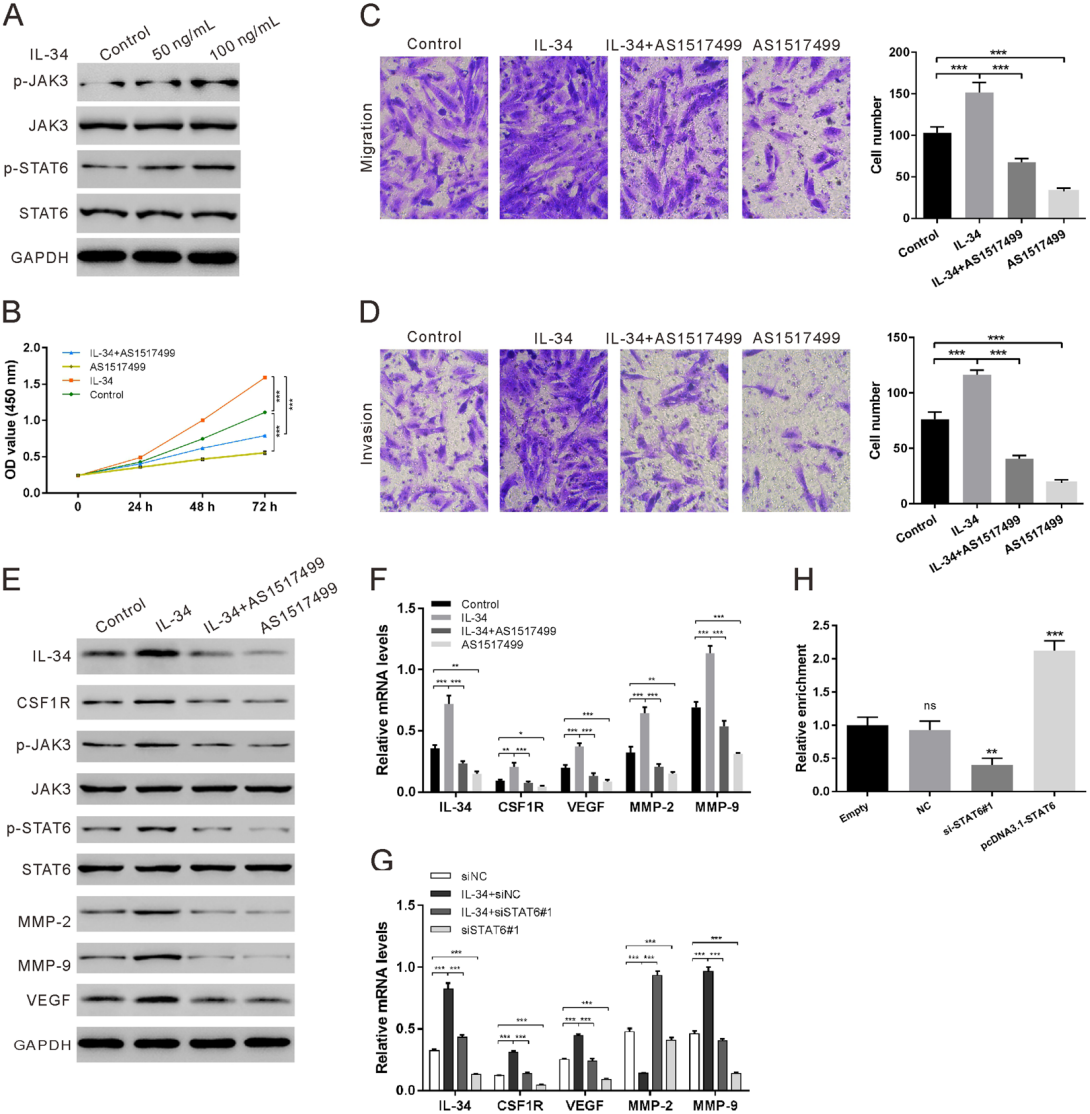
The involvement of CSF1R/JAK3/STAT6 activation and IL-34 autocrine production are responsible for the increases in proliferation, migration and invasion of eutopic ESCs in endometriosis. Eutopic ESCs were isolated from five individuals and three replicates were taken for each experiment. Representative results are shown. (**A**) Western blot showing the screening of related proteins that respond to recombinant IL-34 stimulation. Three independent experiments were performed and representative images are shown. (**B**–**F**) Eutopic ESCs were pre-treated with 100 nM AS1517499 for 30 min and then treated with or without IL-34 (100 ng/mL). Cell proliferation (**B**) detected by CCK-8. Cell migration (**C**) and invasion (**D**) detected by transwell assays. Scale bar: 100 μm. The protein levels of several mediators (**E**) as detected by western blot. Three independent experiments were performed and representative images are shown. The transcriptional levels of several mediators (**F**) as detected by qRT-PCR. (**G**) Eutopic ESCs were treated with STAT6 siRNA (siSTAT6#1)/control siRNA (siNC) in the presence of IL-34 (100 ng/mL). The transcriptional levels of several mediators as detected by qRT-PCR. (**H**) After transfection with si-STAT6 or pcDNA3.1-STAT6, a ChIP assay was used to detect the binding of STAT6 to the IL-34 promoter in ESCs. Immunoprecipitation was performed using anti-STAT6 antibody. Rabbit IgG was used as a negative control. ns: no statistical difference, *P < 0.05, **P < 0.01, ***P < 0.001.

An early study demonstrated that STAT3 was involved in mediating the effect of IL-34^20^. We performed a screening for the potential downstream mediators of IL-34 by western blot analysis with antibodies for the JAK/STAT pathway. Recombinant IL-34 facilitated the phosphorylation and activation of Janus kinase 3 (JAK3) and STAT6 in a dose-dependent manner (Fig. 3A) but had no effect on the expression of the other JAK and STAT family members (Fig. S2). Moreover, AS1517499, a specific STAT6 inhibitor, antagonized the pro-proliferative effect of IL-34 in eutopic ESCs (Fig. 3B). In addition, IL-34-induced migration and invasion were also abrogated upon inactivation of STAT6 (Fig. 3C,D). Collectively, these data demonstrate that the biological activity of IL-34 in eutopic ESCs is mediated by activation JAK3/STAT6 signaling.

Next, we further dissected the precise molecular mechanism by which IL-34 promoted proliferation, migration and invasion. As IL-34 is an alternative ligand of CSF1R^14,15^, CSF1R expression increased with the IL-34 stimulation (Fig. 3E). In addition, the IL-34-induced phosphorylation of JAK3/STAT6 was suppressed by AS1517499 (Fig. 3E). Moreover, IL-34 promoted the transcription and translation of VEGF, MMP-2 and MMP-9, and this alteration was abrogated by treatment with AS1517499 (Fig. 3E,F). Interestingly, we also observed an increased transcription and translation of IL-34 after IL-34 treatment, which was suppressed by AS1517499 (Fig. 3E,F). Thus, we hypothesized that JAK3/STAT6 signaling might regulate IL-34 expression. To test whether JAK3/STAT6 signaling regulates IL-34 expression, STAT6 knockdown and overexpression plasmids were constructed to modulate the expression of STAT6 (Fig. S3). Silencing of STAT6 also showed the same effect as AS1517499 on the mRNA levels of related genes (Fig. 3G). A ChIP assay revealed the presence of an IL-34 promoter sequence in the precipitated complexes (Fig. 3H). Overall, the results suggest that activated STAT6 binds to the promoter of IL-34 to regulate its expression in ESCs. In summary, STAT6 mediated autocrine production of IL-34 triggers the CSF1R/JAK3/STAT6 signaling cascade, which, in turn, up-regulates VEGF, MMP-2 and MMP-9 to promote the pathological progression of ESCs.

### Anti-IL-34 suppresses the proliferation, migration and invasion of ectopic ESCs

**Figure 4:**
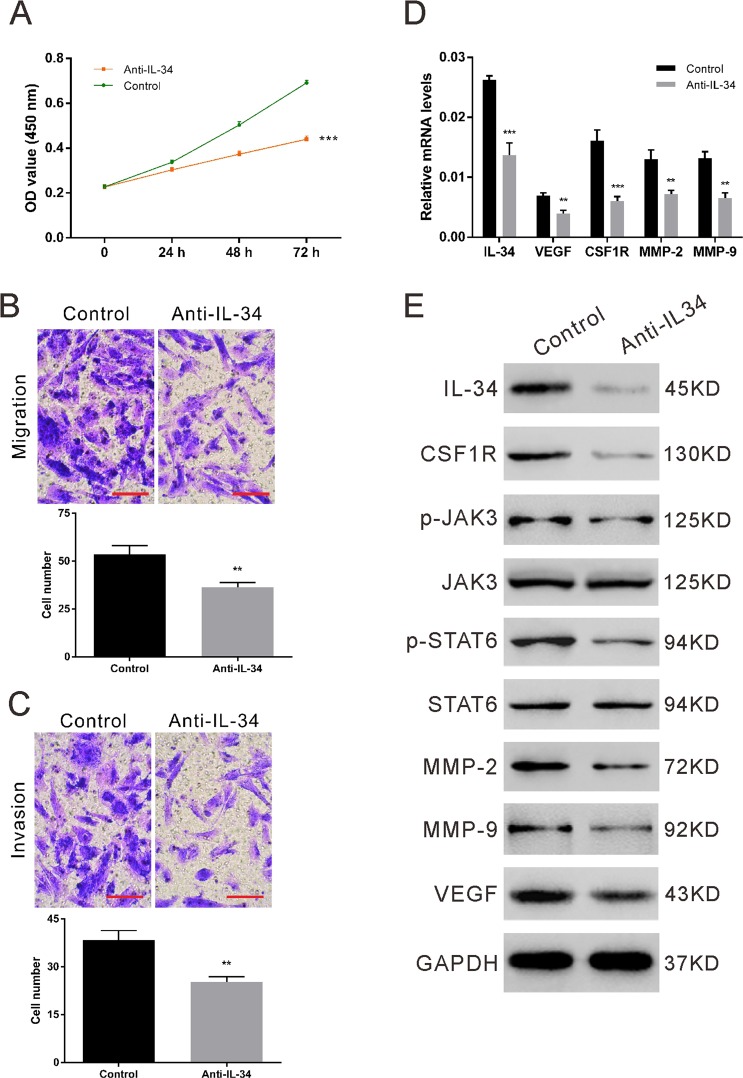
Anti-IL-34 suppresses the proliferation, migration and invasion of ectopic ESCs. Ectopic ESCs were isolated from five individuals and three replicates were taken for each experiment. Representative results are shown. Ectopic ESCs were treated with IL-34 antibody. Cell growth (**A**) as examined by CCK-8 assay. Cell migration (**B**) and invasion (**C**) as examined by transwell assays. Scale bar: 100 μm. The relative mRNA levels of several pivotal molecules (**D**) after normalization to GAPDH. The protein levels (**E**) of above molecules were assessed by western blot analysis. Three independent experiments were performed and representative images are shown. **P < 0.01, ***P < 0.001.

Ectopic ESCs were isolated and pretreated with IL-34 neutralizing antibody to validate the effects of IL-34 in endometriosis. IHC staining showed that ectopic ESCs were successfully isolated (Fig. S4). With IL-34 depletion, the viability and metastatic potential of ectopic ESCs were significantly impaired (Fig. 4A–C). Mechanistically, the depletion of endogenous IL-34 attenuated CSF1R/JAK3/STAT6 activation and down-regulated VEGF, MMP-2 and MMP-9 (Fig. 4D,E), resulting in the altered cellular processes of ectopic ESCs. These results indicate that targeting IL-34 might be an effective therapeutic strategy for the treatment of endometriosis.

### A STAT6 specific inhibitor suppresses endometriosis *in vivo*

**Figure 5:**
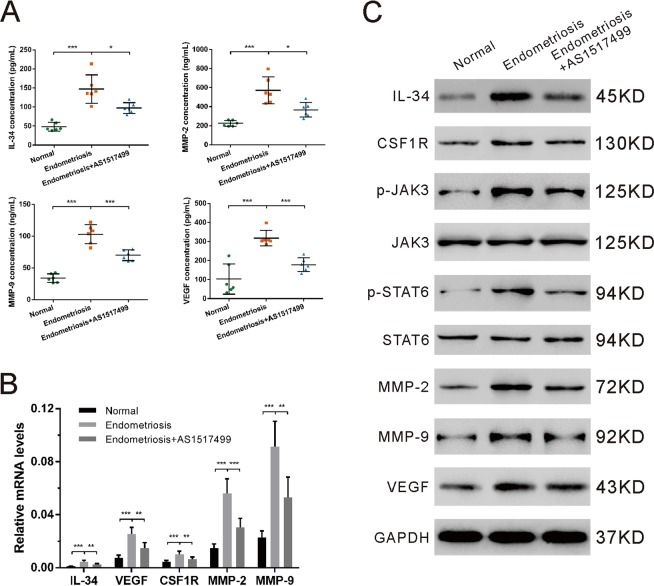
STAT6 signal inhibition with a specific inhibitor suppresses endometriosis *in vivo*. An endometriosis rat model was established to confirm the results *in vitro*. Three groups were divided (n = 6 per group). Normal: rats without endometriosis. Endometriosis: experimental endometriosis rats. Endometriosis + AS1517499: experimental endometriosis rats intraperitoneally injected with AS1517499 (0.1 mg/kg) once a day. (**A**) The concentrations of IL-34, VEGF, MMP-2 and MMP-9 in the sera of rats. (**B**) The mRNA levels of IL-34, CSF1R, VEGF, MMP-2 and MMP-9 in endometrial tissue and eutopic implants. (**C**) The protein levels of related molecules in endometrial tissue and eutopic implants were assessed by western blot analysis. Three independent experiments were performed and representative images are shown. *P < 0.05, **P < 0.01, ***P < 0.001.

An endometriosis rat model was established, as previously described^25^, to validate the essential role of the IL-34/CSF1R/JAK3/STAT6 signaling pathway *in vivo*. Endometriotic lesions harvested from the Endometriosis + AS1517499 group appeared smaller than those from Endometriosis group (Fig. S5). Unsurprisingly, IL-34, VEGF, MMP-2 and MMP-9 were increased in the sera of the experimental endometriosis rats, which could be abrogated by STAT6 signaling blockage with AS1517499 (Fig. 5A). Eutopic endometrial tissues and ectopic implants were also collected for qRT-PCR and western blot analysis. The transcription and translation of IL-34, CSF1R, VEGF, MMP-2 and MMP-9 were elevated (Fig. 5B,C). Moreover, results revealed that p-JAK3 and p-STAT6 proteins increased in the ectopic implants (Fig. 5C). Inhibition of STAT6 signaling with AS1517499 suppressed the expression of the above proteins in the endometriosis rats (Fig. 5B,C). In general, targeting the IL-34/CSF1R/JAK3/STAT6 pathway with a STAT6 inhibitor was an effective means of treating endometriosis in rats.

## Discussion

In our present study, through reanalysis of the expression profile of IL-34 in the microarray data of the Gene Expression Omnibus (GEO) database (GSE51981), we found that IL-34 was increased in clinical samples with endometriosis. IL-34 plays a critical role in cell proliferation, differentiation, apoptosis, angiogenesis, inflammation and immunoregulation^18,26^. Numerous diseases are attributed to the dysregulation of IL-34 signaling^27–29^. Thus, we hypothesized that IL-34 may play an important role as a multifunctional inflammatory cytokine in the pathogenesis and persistence of endometriotic lesions.

First, we showed that excessive secretion of IL-34 was detected in the sera of endometriosis patients and in rat models of endometriosis. Second, CSF1R, p-JAK3, p-STAT6, VEGF, MMP-2 and MMP-9 were up-regulated in response to recombinant IL-34 in *vitro*. VEGF, MMP-2 and MMP-9 are closely related to the pathogenesis of endometriosis^21–23^. According to our study, recombinant IL-34 promoted the expression of these proteins in eutopic ESCs, which indicates that IL-34 may participate in several important cellular processes that are dysregulated in ESCs, such as proliferation, migration and invasion. Third, a chromatin immunoprecipitation (ChIP) assay confirmed the binding of STAT6 to the IL-34 promoter. AS1517499, an inhibitor of STAT6, remarkably abrogated the cellular alterations induced by IL-34. As a transcription factor of the STAT family, STAT6 is implicated in the pathology of a wide variety of diseases^30–32^. A genetic polymorphism analysis demonstrated the association between STAT6 and endometriosis^33^. The present study suggests that IL-34 is also an activator of STAT6 signaling by binding to CSF1R in ESCs. Further, an IL-34 neutralizing antibody could attenuate CSF1R/JAK3/STAT6 activation and down-regulate VEGF, MMP-2 and MMP-9, eventually leading to suppression of the development of endometriosis *in vitro*. These results were further validated *in vivo* through endometriosis lesions in a rat model. Taken together, these results indicate that IL-34 facilitates endometriosis progression by promoting ESC proliferation, migration and invasion.

Based on previous reports^34–36^, we chose IL-34 concentrations of 0–200 ng/mL for the functional assays. We found that IL-34 concentrations of 25 to 100 ng/mL promoted eutopic ESC growth in a dose-dependent manner. Here, the doses of IL-34 for *in vitro* experiments were much higher than the level of IL-34 in serum samples from endometriosis patients, which were typically less than 400 pg/mL. The possible reason was that the IL-34 concentration surrounding ESCs could be higher than the serum concentration of Il-34.

Because CSF1R is not an exclusive receptor for IL-34, IL-34 may exert pathophysiological effects through other receptor molecules. Receptor-type protein-tyrosine phosphatase ζ (PTPRZ1) was recently identified as an additional IL-34 receptor^37^. IL-34 binds to the extracellular domain of PTPRZ1 to stimulate the phosphorylation of paxillin and focal adhesion kinase, which influences the growth and migration of glioblastoma cells. Whether CSF1R is necessary and sufficient for the activation of JAK3/STAT6 signals induced by IL-34 needs further investigation. In addition, CSF-1 is structurally and functionally analogous to IL-34 and its effects are mediated exclusively through CSF1R^15^. Mounting evidence suggests that CSF-1 is increased in endometriosis and is conducive to the formation of endometriotic lesions^38,39^. Whether CSF-1 is involved in the activation of CSF1R/JAK3/STAT6 signaling is still unclear. Moreover, the recruitment of immune cells in the endometriotic microenvironment^40^ may also influence the production of IL-34 and deserves further investigation.

In conclusion, our research reveals the functional role of IL-34 in endometriosis as well as the mechanism by which IL-34 mediates its effects. With this study, we hope to provide insight for the development of novel therapeutic strategies in endometriosis.

## Materials and Methods

### Ethical approval

The use of human samples was approved by the Ethics Committee of Women’s Hospital, Zhejiang University School of Medicine (Hangzhou, China) (No. 2019-005). All animal experiments were approved by the Animal Care and Use Committee of Women’s Hospital, Zhejiang University School of Medicine (Hangzhou, China). All research was performed in accordance with the relevant guidelines and regulations.

### Reanalysis of the Gene Expression Omnibus (GEO) dataset

The endometriosis dataset, which contains microarray data of endometrial specimens from women with Non-Endometriosis and No Uterine P/pelvic pathology, women with Non-Endometriosis but with Uterine P/pelvic pathology, women with Minimal/Mild endometriosis and women with Moderate/Severe endometriosis, was downloaded from the GEO database using accession number GSE51981^20^, and the expression of IL-34 was compared using an one-way ANOVA analysis followed by Tukey’s test.

### Clinical samples

A total of 90 endometriosis patients (age range 25–43, M ± SD. 35.2 ± 5.7) and 90 non-endometriosis patients (age range 26–48, M ± SD. 36.6 ± 4.8) admitted at Women’s Hospital, Zhejiang University School of Medicine were enrolled in this study after written informed consent was obtained. The endometriosis patients were at stage III–IV (according to revised AFS classification) (23cases of stage III/67 cases of stage IV), and 75.6% (68 of 90) of the patients had chronic pelvic pain and 41.1% (37 of 90) had infertility. The non-endometriosis patients included 36 (40.0%) cases of mature teratoma and 54 (60.0%) cases of tubal infertility. There was no difference in body mass index (BMI) between the two groups. The samples were all obtained during the proliferative phase of the menstrual cycle. The menstrual cycle phase was determined by preoperative history and histological examination. The inclusion criteria were as follows: regular menstruation with a period of 28 to 32 days; no other endocrine, immune and metabolic diseases, no hormones or other medications were received within three months before surgery; non-lactating; and no history of serious drug allergies. The sera were collected from all the participants and stored at −80 °C until ELISA analysis. The eutopic endometrial samples and the non-fibrotic red ectopic endometria were collected during laparoscopic excision of endometrioma and used for the isolation of eutopic and ectopic endometrial stromal cells (ESCs), respectively.

### Cell isolation and cell culture

Primary cells were isolated as previously described^41^. Tissue samples were washed with PBS and cut into pieces. The tissue pieces were digested with 0.125% collagenase IV (Sigma, St. Louis, MO, USA) for 60 min and centrifuged at 500 rpm for 5 min. Then, the cell suspension was collected and centrifuged at 3000 rpm for 10 min. Cell pellets were resuspended in DMEM/F12 medium (Hyclone, SH30023.01B; Logan, UT, USA) supplemented with 10% fetal bovine serum (Gibco, 16000-044; Carlsbad, CA, USA) and 1% penicillin-streptomycin (Solarbio, Beijing, China) and incubated at 37 °C with 5% CO_2_. After 2 h, ESCs attached to surface of the plate, and the culture medium was replaced to discard the immunocytes and epithelial glands. The isolated cells were identified by IHC staining with anti-CK19 and anti-CD10 antibodies (Supplemental Table 2).

### Enzyme linked immunoabsorbent assay (ELISA)

The concentrations of IL-34, MMP-2, MMP-9 and VEGF in the sera were detected by ELISA according to the manufacturer’s instructions (X-Y Biotechnology, Shanghai, China). Briefly, the prepared standard samples and serum samples were added to the wells coated with the specific antibody and incubated at 37 °C. Then the liquids were discarded and the wells were washed with wash buffer. Horseradish peroxidase-conjugate reagent was added to each well except the blank group. After incubation and another wash, tetramethylbenzidine (TMB) substrate solution was added and the plate was incubated in dark. Finally, the reaction was terminated by addition of a stop solution and the absorbance at 450 nm was measured.

### Cell counting kit-8 (CCK-8) assay

CCK-8 assay was performed using a Cell Proliferation and Cytotoxicity Assay Kit (SAB, CP002; College Park, MD, USA). Briefly, 100 μL of cell suspension containing 2 × 10^3^ ESCs was added to each well of the 96-well plates. After incubating overnight, the cells were divided into different groups and exposed to different treatments. Finally, 10 μL of CCK-8 solution was added to each well. Cell viability was evaluated by measuring the absorbance at 450 nm.

### Transwell assays

Transwell inserts (Corning, 3422) were used to detect cell migration and invasion. Cultured ESCs were trypsinized and resuspended in serum-free DMEM/F12 medium, then seeded into 6-well plates and treated with recombinant 50 ng/mL or 100 ng/mL human IL-34 (R&D Systems, 5265-IL; Minneapolis, MN, USA), 100 nM AS1517499 (a specific STAT6 inhibitor, Medchemexpress, HY-100614; Monmouth Junction, NJ, USA) or 50 ng/mL IL-34 antibody (Abcam, ab101443; Cambridge, MA, USA) for 48 h. For invasion assays, 80 µL of matrigel (Corning, 356234; New York, NY, USA) was added to the upper chamber in advance. 0.3 mL of an ESC cell suspension containing 9 × 10^4^ cells was added to each insert and different groups were divided according to the treatment. 0.7 mL of complete DMEM/F12 medium was added to the lower chamber. After culturing for 24 h, the inserts were doused with 4% paraformaldehyde and 0.5% crystal violet solution, successively. Cells were counted under a light microscope.

### Plasmid construction and cell transfection

Signal transducer and activator of transcription 6 (STAT6, NM_001178078.1) interference sequences (Position 547–569: 5′-GCACCAUCUUGCAACACAUTT-3′. Position 1673–1695: 5′-GAUGUGUGAAACUCUGAACTT-3′. Position 2451–2473: 5′-GAAUCAGUCAACGUGUUGUTT-3′) were cloned into a plasmid to knockdown STAT6 expression. The coding sequence of STAT6 was synthesized using the primers containing the restriction enzyme cutting sites of Hind III and EcoRI (Forward: 5′-CCCAAGCTTATGTCTCTGTGGGGTCTGGTCTCC-3′; Reverse: 5′-CCGGAATTCCCAACTGGGGTTGGCCCTTAGG-3′) and integrated into pcDNA3.1(+) to elevate STAT6 expression. Eutopic ESCs were transfected with the STAT6 knockdown or overexpression vectors. Cell transfection was performed with Lipofectamine 2000 (Invitrogen, Carlsbad, CA, USA) according to the manufacturer’s instructions.

### Quantitative real-time PCR (qRT-PCR)

mRNA levels were detected by qRT-PCR. RNA samples were reverse transcribed to cDNA using the RevertAid First Strand cDNA Synthesis Kit (Fermentas, Hanover, MD, USA) and amplified with the SYBR Green qPCR Master Mixes (Thermo Fisher, Rockford, IL, USA) according to the manufacturers’ instructions. The mRNA levels were estimated using the 2^−ΔCt^ method after normalization to GAPDH. The primer sequences of detected genes for qRT-PCR are listed in Supplemental Table 1.

### Western blot analysis

Protein levels were detected by western blot analysis. Briefly, the lysates were separated by gel electrophoresis. Proteins were then transferred onto polyvinylidene fluoride membranes and blocked with 5% nonfat milk. The membranes were then incubated with optimally diluted primary antibodies and second antibodies sequentially. Protein expression was assessed with a chemiluminescent imaging system (Tanon 5200, Shanghai, China). The antibodies used for Western blot are listed in Supplemental Table 2.

### Chromatin immunoprecipitation assay (ChIP)

After transfection with si-STAT6 or pcDNA3.1-STAT6, eutopic ESCs were cross-linked with 1% formaldehyde. Then the cells were lysed and chromatin was sheared to DNA fragments of 150–900 bp by sonication. Immunoprecipitation was performed using an anti-STAT6 antibody. Rabbit IgG was used as a negative control. The precipitated complexes were washed and reverse cross-linked. After purification, the extracted DNA was amplified by qRT-PCR to identify the presence of an IL-34 promoter sequence. The primers which were specific for the IL-34 promoter that contained STAT6 binding site are listed as follows. Forward: 5′-GGTTGAAGACTCCCTCCTAC-3′. Reverse: 5′-AAAGCAGGCCACTGCAGCTC-3′.

### Endometriosis rat model and *in vivo* study

Eighteen female wistar rats weighting 210–240 g were obtained to establish the endometriosis model. The rats accepted preoperative diethylstilbestrol injection with a dose of 0.1 mg/kg. Rat surgeries were conducted as previously described^25^. Briefly, a vaginal secretion smear was applied to confirm the estrus cycle. After anesthetization with chloral hydrate (0.4 mL/100 g), a right abdominal incision was made to isolate the right uterine horn. Then the uterine horn was excised. The stripped endometrium was cut into fragments and planted in the abdominal cavity and subcutis. Penicillin was applied to prevent infection. The rats were divided into three groups (n = 6 per group) according to the treatment: Normal, rats underwent laparotomy surgery without the transplant of endometrial tissues; Endometriosis, experimental endometriosis rats; Endometriosis + AS1517499, experimental endometriosis rats intraperitoneally injected with AS1517499. The rats that underwent laparotomy surgery without the transplant of endometrial tissues were used as a control.. Then, exploratory laparotomy was performed to check the formation of ectopic lesions after two weeks, which indicated the successful establishment of an endometriosis rat model. The endometriosis rats were then intraperitoneally injected with AS1517499 (0.1 mg/kg) once a day. Ten weeks later, the rats were sacrificed. Peripheral blood, endometrial tissue and eutopic implants were collected for further examination.

### Statistical analysis

GraphPad Prism 6 software (San Diego, CA, USA) was used for statistical analysis. At least three independent experiments were performed for each result. Data are presented as mean value ± SD. One-way ANOVA and student’s *t* test were applied for the comparison of mean values. P < 0.05 was regarded as statistically significant.

## Supplementary information

Supplementary Tables & Figures
