# Supplementary material for: Autocrine Production of Interleukin-34 Promotes the Development of Endometriosis through CSF1R/JAK3/STAT6 signaling
Source: Sci Rep. 2019 Nov 14;9:16781. doi: 10.1038/s41598-019-52741-1 (PMC6856158; doi:10.1038/s41598-019-52741-1)
Supplement: Supplementary file 1 — Supplementary Tables & Figures [file 41598_2019_52741_MOESM1_ESM.docx]

**Autocrine Production of Interleukin-34 Promotes the Development of Endometriosis through CSF1R/JAK3/STAT6 signaling**

Kaiqing Lin ^a^, Junyan Ma ^b^, Yaomin Peng ^b^, Meina Sun ^a^, Kaihong Xu ^a^, Ruijin Wu ^a^,

and Jun Lin ^a,*^

^a^ Department of Gynecology, Women’s Hospital, Zhejiang University School of Medicine, 1 Xueshi Road, Hangzhou, Zhejiang, People’s Republic of China, 310006

^b^ Key Laboratory of Reproductive Genetics (Zhejiang University), Ministry of Education, People’s Republic of China, 310006

^*^Corresponding author: Dr. Jun Lin, Department of Gynecology, Women’s Hospital, Zhejiang University School of Medicine, 1 Xueshi Road, Hangzhou, Zhejiang, People’s Republic of China, 310006 (Phone: 86-571-87061501; Fax: 86-571-87061878; E-mail: linjun@zju.edu.cn).

Supplemental Table 1. Primer sequences used in the study

| **Species** |  | **Gene symbol** | **Primer sequence** |
| --- | --- | --- | --- |
| Homo sapiens |  | STAT6 (NM_001178078.1) | F 5’-GCCAAAGCCCTAGTGCTGAA-3’  R 5’-GACGAGGGTTCTCAGGACTTC-3’ |
|  |  | IL34 (NM_001172771.1) | F 5’-AAGGTGGAATCCGTGTTGTCC-3’  R 5’-AGCTTTGTTTACAGCAGGAGC-3’ |
|  |  | CSF1R (NM_001288705.2) | F 5’-TCCAAAACACGGGGACCTATC -3’  R 5’-CGGGCAGGGTCTTTGACATA-3’ |
|  |  | MMP2 (NM_001127891.2) | F 5’-TTGGTGGGAACTCAGAAG-3’  R 5’-TTGCGGTCATCATCGTAG-3’ |
|  |  | MMP9 (NM_004994.2) | F 5’-AAGGGCGTCGTGGTTCCAACTC-3’  R 5’-AGCATTGCCGTCCTGGGTGTAG-3’ |
|  |  | VEGF (NM_001025366.2) | F 5’-GACAGATCACAGGTACAG-3’  R 5’-GAAGCAGGTGAGAGTAAG-3’ |
|  |  | GAPDH (NM_001256799.1) | F 5’-CACCCACTCCTCCACCTTTG-3’  R 5’-CCACCACCCTGTTGCTGTAG-3’ |
| Rattusnorvegicus |  | IL-34 (NM_001025766.1) | F 5’-GCAGTCGGGAGAAACCAGAG-3’  R 5’-AGGAGGAGGAAGAGGGAGAG-3’ |
|  |  | CSF1R (NM_001029901.1) | F 5’-GACCACCATCCACATCTACC-3’  R 5’-CACTGCCATTGCTCACAC-3’ |
|  |  | MMP2 (NM_031054.2) | F 5’-ACCAAGAACTTCCGACTATCC-3’  R 5’-CTGAGCAATGCCATCAAAGAC-3’ |
|  |  | MMP9 (NM_031055.1) | F 5’-TCTCTACTGGGCATTAGGG-3’  R 5’-GTGTCCGAGGAAGATACTTG-3’ |
|  |  | VEGF (NM_001110333.2) | F 5’-CCAAAGCCAGCACATAGG-3’  R 5’-TCTCCGCTCTGAACAAGG-3’ |
|  |  | GAPDH (NM_017008.4) | F 5’-GGAGTCTACTGGCGTCTTCAC-3’  R 5’-ATGAGCCCTTCCACGATGC-3’ |

Supplemental Table 2. Antibodies used in the study

| **Protein symbol** | **Manufacturer** | **Catalog #** | **Dilution factor** |
| --- | --- | --- | --- |
| JAK1 | abcam | Ab133666 | 1:1000 |
| p-JAK1 | abcam | Ab138005 | 1:500 |
| p-JAK2 | CST | #8082 | 1:1000 |
| JAK2 | CST | #3230 | 1:1000 |
| p-JAK3 | sigma | SAB4504249 | 1:1000 |
| JAK3 | abcam | Ab203611 | 1:500 |
| p-STAT3 | abcam | Ab76315 | 1:5000 |
| STAT3 | CST | #9139 | 1:2000 |
| STAT4 | abcam | Ab68156 | 1:1000 |
| p-STAT4 | abcam | Ab28815 | 1:800 |
| p-STAT5a | abcam | Ab30648 | 1:1000 |
| STAT5a | abcam | Ab32043 | 1:1000 |
| STAT5b | abcam | Ab178941 | 1:500 |
| p-STAT5b | abcam | Ab52211 | 1:5000 |
| p-STAT6 | abcam | Ab28829 | 1:800 |
| STAT6 | abcam | Ab44718 | 1:200 |
| IL-34 | abcam | Ab74548 | 1:2000 |
| CSF1R | Santa Cruz | Sc-70451 | 1:500 |
| MMP-2 | abcam | Ab14311 | 1:2000 |
| MMP-9 | abcam | Ab38898 | 1:2000 |
| VEGF | abcam | Ab46154 | 1:1000 |
| GAPDH | CST | #5174 | 1:2000 |
| CK19 | abcam | Ab7754 | 1:100 |
| CD10 | abcam | Ab227659 | 1:200 |


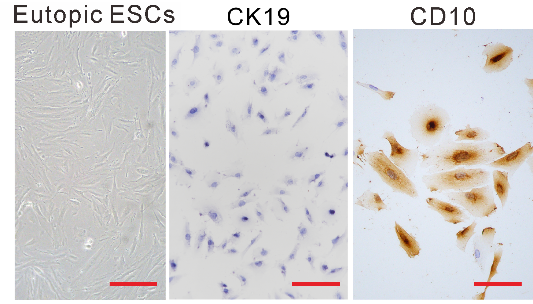


Figure S1. Eutopic ESCs were derived from endometriosis patients and identified by IHC straining of CK19 and CD10. Scale bar: 100 μm.


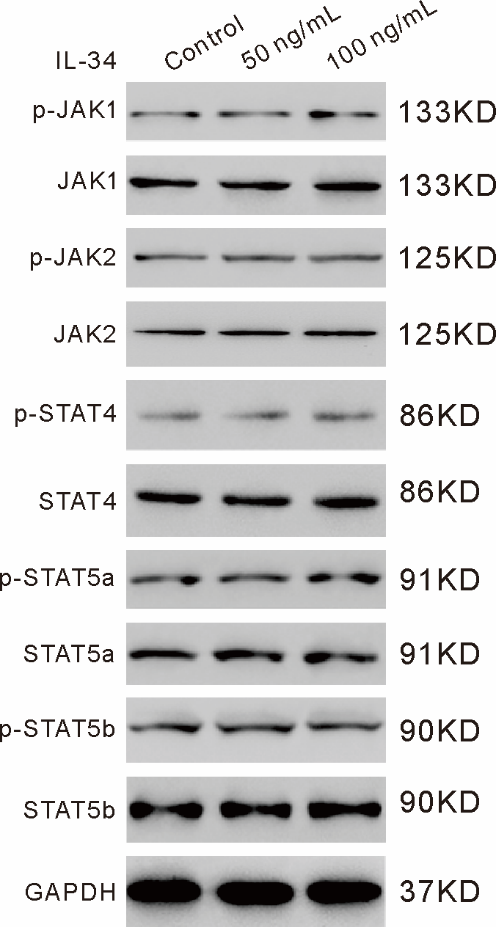


**Figure S2.** The screening of related proteins that responded to recombinant IL-34 stimulation by western blot.


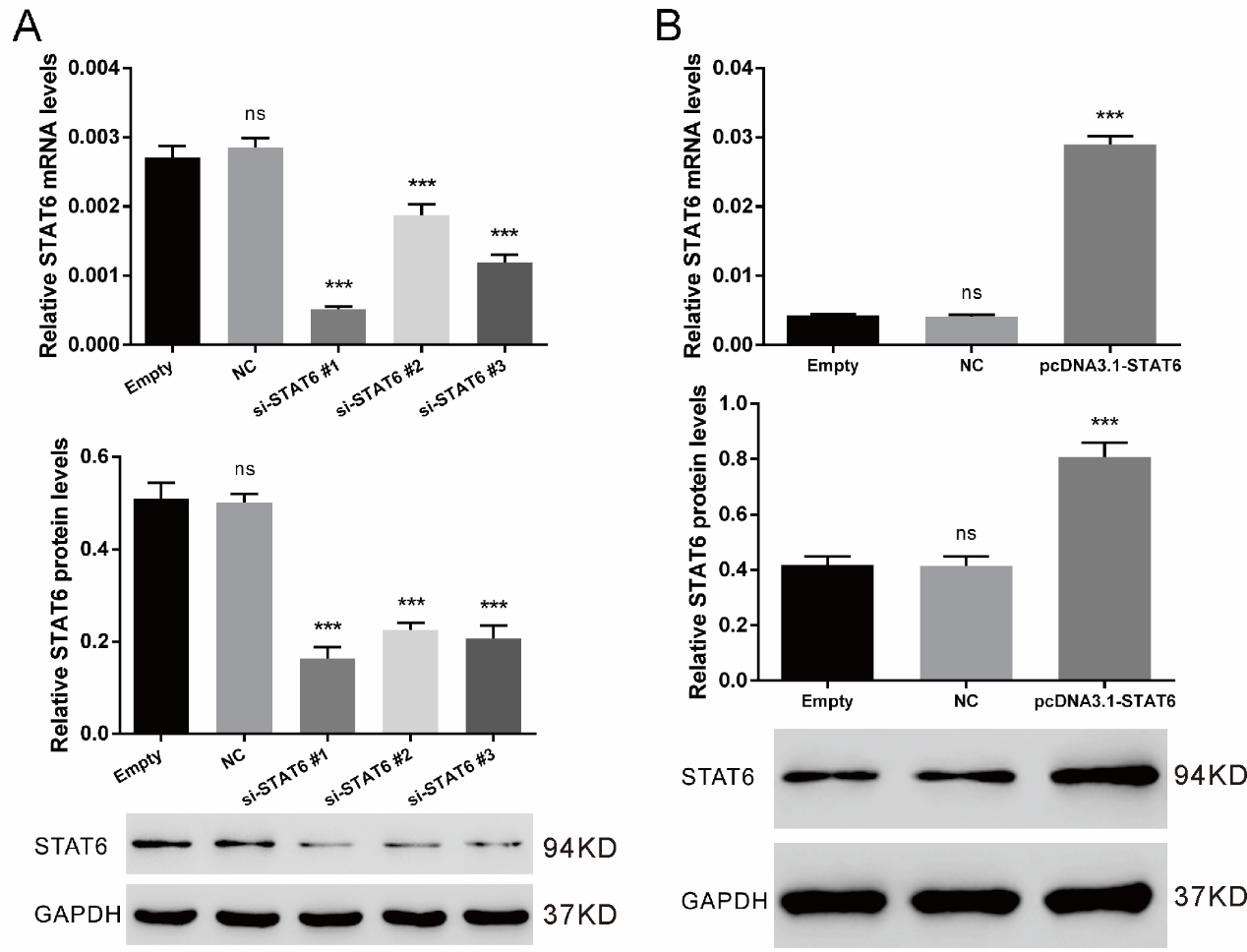


**Figure S3.** Manipulation of STAT6 expression in eutopic ESCs. (A) STAT6 silencing in ESCs after transfected with si-STAT6 was confirmed by qRT-PCR and western blot. (B) STAT6 over-expression in ESCs after transfected with pcDNA3.1-STAT6 was verified by qRT-PCR and western blot. ns: no statistical difference, *** P<0.001.


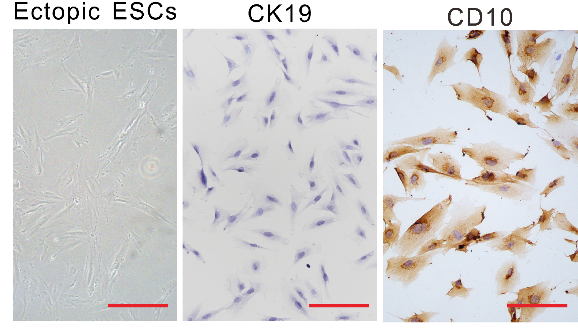


**Figure S4.** Ectopic ESCs were separated from ectopic endometriotic lesions and identified by IHC staining of CK19 and CD10. Scale bar: 100 μm.


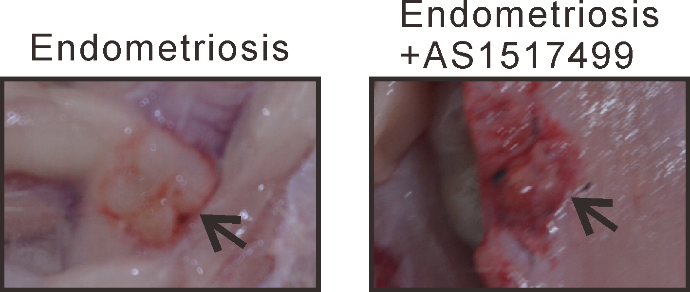


**Figure S5.** STAT6 signaling blockage with specific inhibitor suppressed endometriosis *in vivo*. Endometriotic lesions harvested from Endometriosis+AS1517499 group appeared smaller than those from Endometriosis group. Arrow indicate the endometriotic lesion.
